# Patient outcomes from a physical activity programme for cancer survivors in general practice: an intervention implementation study

**DOI:** 10.3399/BJGP.2024.0558

**Published:** 2025-04-23

**Authors:** Famke Huizinga, Nico-Derk L Westerink, Annemiek ME Walenkamp, Annette J Berendsen, Mathieu HG de Greef, Michiel R de Boer, Geertruida H de Bock, Marjolein Y Berger, Daan Brandenbarg

**Affiliations:** Department of Primary and Long-term Care, University Medical Center Groningen, University of Groningen, Groningen.; Department of Primary and Long-term Care, University Medical Center Groningen, University of Groningen, Groningen.; Department of Medical Oncology, University Medical Center Groningen, University of Groningen, Groningen.; Department of Primary and Long-term Care, University Medical Center Groningen, University of Groningen, Groningen.; Department of Human Movement Sciences, University Medical Center Groningen, University of Groningen, Groningen.; Department of Primary and Long-term Care, University Medical Center Groningen, University of Groningen, Groningen.; Department of Epidemiology, University Medical Center Groningen, University of Groningen, Groningen.; Department of Primary and Long-term Care, University Medical Center Groningen, University of Groningen, Groningen.; Department of Primary and Long-term Care, University Medical Center Groningen, University of Groningen, Groningen.

**Keywords:** cancer survivors, exercise, general practice, implementation science, outcome assessment, primary health care

## Abstract

**Background:**

Physical activity (PA) benefits cancer survivors’ health, yet no PA programmes are incorporated in Dutch general practice.

**Aim:**

To evaluate cancer survivors’ outcomes following a PA programme in general practice.

**Design and setting:**

A single-arm PA intervention implementation study among cancer survivors in 15 Dutch general practices.

**Method:**

Patients aged ≥18 years who completed primary cancer treatment ≥6 months prior were eligible. The 9-month intervention comprised counselling sessions with a primary care practitioner (PCP) aimed at increasing daily PA. Reach, Effectiveness, and Implementation of the RE-AIM framework were evaluated among participants. Primary health outcomes included self-reported symptoms of fatigue, depression, and anxiety; secondary outcomes included step count, caloric expenditure, weight, physical function, self-reported quality of life, and PA. Outcomes were assessed at time (T)_0_–T_3_ (0, 3, 6, and 9 months) or at PCPs’ sessions S1–S6 (0, 3, 6 weeks, and 3, 6, 9 months). Non-participants completed a single baseline questionnaire. The study used (non-)parametric independent tests and linear mixed models for analyses.

**Results:**

Of 564 invited patients, 149 (26%) participated. Participants had less formal education, higher unemployment, less PA, and more fatigue and psychological symptoms than non-participants. All primary and most secondary health outcomes improved over time, with clinically relevant changes in step count and physical function. In total, 11% (*n* = 16/149) dropped out before and 26% (*n* = 35/133) during the programme. Counselling session adherence and PA goal achievement were 98% (*n* = 647/661) and 73% (*n* = 81/111), respectively.

**Conclusion:**

The programme reached long-term cancer survivors with poorer health status, and showed positive health changes particularly on PA and physical function. Such PA programmes may benefit the health of a rising number of cancer survivors visiting primary care.

## Introduction

Cancer poses a significant health challenge, especially in Western countries. Europe accounts for nearly a quarter of patients with cancer despite having just 10% of the worldwide population.^1^ Owing to increased survival rates, more cancer survivors live with the consequences of diagnosis and treatment, with fatigue (∼40%), depression, and anxiety (∼21%) as the most prevalent long-term symptoms, with these having an impact on quality of life.^2^^–^^6^

Lifestyle interventions have received increased attention as treatment for these symptoms, with physical activity (PA) interventions among the most studied.^7^^–^^9^ There is ample scientific evidence that PA can significantly reduce fatigue, depression, and anxiety,^10^^–^^15^ and it might even surpass psychological or pharmaceutical treatments in effectiveness for fatigue.^16^

Despite this evidence and guidelines recommending PA interventions since 2010,^17^^,^^18^ implementation remains challenging. Participation rates are generally low,^19^^–^^22^ often missing those who could benefit the most. Participants typically have more formal education, are non-smokers, consume less alcohol, have higher PA levels, and lower psychological distress.^23^^–^^25^ PA levels among cancer survivors vary; studies indicate lower levels compared with healthy controls,^4^^,^^26^^,^^27^ whereas others suggest higher levels, depending on cancer diagnosis.^27^^–^^29^ This highlights the need for personalised, accessible care, and research on effective implementation.^4^^,^^30^^,^^31^

In countries with robust, fully covered primary care like the Netherlands, primary care practitioners (PCPs) are well-positioned to provide lifestyle care for cancer survivors.^32^^–^^34^ GPs and practice nurses, especially, can reach a broad population owing to their accessibility without requiring out-of-pocket deductibles or additional insurance. They maintain long-standing relationships with their patients and are gatekeepers to further care.^33^^,^^35^^–^^37^ Also, practice nurses are responsible for managing other lifestyle-related conditions, which may facilitate the integration of this care.^38^ Despite their potential, implementation of PA programmes for cancer survivors in a pressurised primary care setting are limited, with little known about participants’ outcomes. This study implemented a PA programme in Dutch general practice for cancer survivors, and evaluated patient outcomes within ‘Reach’, ‘Effectiveness’, and ‘Implementation’ of the RE-AIM framework. The RE-AIM framework is widely used for evaluating the implementation of health interventions and PA programmes in particular.^39^

**Table:** How this fits in

With increasing survival rates, GPs see more cancer survivors with long-term symptoms of fatigue, depression, and anxiety. Physical activity (PA) is known to alleviate these symptoms, but no PA programmes have been incorporated into Dutch GP care. Yet the GP practice can play a pivotal role in lifestyle care and management of chronic symptoms. This research aimed to evaluate patient outcomes following the implementation of a PA programme among cancer survivors in general practice. A home-based PA programme, with counselling and activity tracker use, reached cancer survivors with poorer health status and showed favourable health changes particularly on PA and physical function. Such PA programmes may benefit the health of a rising number of cancer survivors visiting primary care.

## Method

### Study design

This was a single-arm PA intervention implementation study in 15 Dutch general practices from December 2020 to June 2024. Details of design and procedures are published elsewhere.^40^

### Study population

Patients aged ≥18 years who completed primary cancer treatment ≥6 months prior were eligible, including those undergoing adjuvant hormonal therapy. Exclusion criteria included non-melanoma skin cancer, participation in another PA programme, terminal illness, or any physical, cognitive, or other impairments hindering participation judged by their PCP. The PCP, if needed with the help of a researcher, selected all patients with cancer from their electronic patient records and assessed eligibility. Some PCPs also recruited based on opportunity – for example, when patients consulted them or were part of chronic care programmes. Selected patients were invited by their general practice and could either participate in the PA programme (participants) or only complete the baseline time (T)_0_ questionnaire (non-participants).

### Intervention

The intervention was a home-based, counselling protocol of 9 months, consisting of six counselling sessions with a PCP at the patient’s general practice, aimed at increasing daily PA (for example, walking, cycling, and gardening). PCPs were practice nurses in 12 practices, doctors assistants in two, and a dietitian in one. All were trained in the programme protocol.^40^ A Fitbit Charge 3 was used for PA monitoring, goal-setting, and feedback. Sessions were tailored to the patients, but generally the first four sessions were aimed at increasing PA; the final two sessions focused on maintaining the new PA behaviour. General practices were financially compensated for their time dedicated to the project.

### Data collection

The domains of Reach (participation and representativeness), Effectiveness (health outcomes), and Implementation (adherence and goal achievement) of the RE-AIM framework were evaluated at the patient level.^41^ Measurements were collected through self-report questionnaires at T_0_–T_3_ (baseline, 3, 6, and 9 months) or measured during sessions S1–S6 (0, 3, 6 weeks, and 3, 6, 9 months) by the PCP (Box 1).

**Box 1.** An overview of the measures and the measurement instruments used to evaluate the dimensions of the RE-AIM framework

**RE-AIM dimension, measures**	**Measurement instrument**	**Time**
**Reach (R)**		
• R1. Participation rate: participating versus invited	Documentation	T_0_
• R2. Representativeness of participants		
□ a) Demographic information	Questionnaire	T_0_
▪ Age		
▪ Gender		
▪ Level of education		
▪ Employment status		
▪ Living situation		
▪ Care received at home		
□ b) Lifestyle measures		
▪ Smoking behaviour	Questionnaire^ab^	S1/T_0_
▪ Alcohol use	Questionnaire^ab^	S1/T_0_
▪ Motivation for PA	SOC^a^	S1/T_0_
▪ Self-reported PA	IPAQ-SF	T_0_
□ c) Clinical characteristics	General practice records	T_0_
▪ Cancer diagnoses		
▪ Cancer stage	TNM staging	
▪ Time since diagnosis		
▪ Type of treatment		
▪ Time since treatment		
▪ Comorbidities	CCI	
□ d) Health outcomes		
▪ Fatigue	FACT-F	T_0_
▪ Depression	HADS-D	T_0_
▪ Anxiety	HADS-A	T_0_
▪ Quality of Life	FACT-G	T_0_
• R3. Reasons of the PCPs for not inviting	Documentation	
• R4. Reasons for (non-)participation	Questionnaire	

**Effectiveness (E)**		
• E1. Primary health outcomes		
□ Fatigue	FACT-F	T_0_–T_3_
□ Depression	HADS-D	T_0_–T_3_
□ Anxiety	HADS-A	T_0_–T_3_
• E2. Secondary health outcomes		
□ Number of steps	Activity tracker	S1–S6
□ Caloric expenditure	Activity tracker	S1–S6
□ Weight	Scale	S1, S4, S6
□ Lower-limb strength	30s sit-to-stand test	S1, S4, S6
□ Aerobic endurance	2-min step test	S1, S4, S6
□ Self-reported PA	IPAQ short form	T_0_, T_1_, T_3_
□ Quality of life	FACT-G	T_0_–T_3_
• E3. Experiences of PA programme	Questionnaire	T_1_–T_3_
**Implementation (I)**		
• I1. Dropout rate and reasons	PCP or researchers’ documentation	
• I2. Adherence to counselling sessions	Registration by PCP	S1–S6
• I3. Adherence to wearing activity tracker	Questionnaire	T_1_–T_3_
• I4. Accomplishment of PA goals	Registration by PCP	S2–S4

a *For participants, the practice nurse asked about the measure in S1; for non-participants, this was based on self-report at T_0_*.

b *Smoking behaviour was assessed by the number of years smoked, and number of cigarettes smoked per day. Alcohol use was assessed as the number of alcohol units per day. CCI = Charlson Comorbidity Index. FACT-F = Functional Assessment of Cancer Therapy — Fatigue. FACT-G = Functional Assessment of Cancer Therapy — General. HADS-A = Hospital Anxiety and Depression Scale — Anxiety. HADS-D = Hospital Anxiety and Depression Scale — Depression. IPAQ-SF = International Physical Activity Questionnaire — Short Form. PA = physical activity. PCP = primary care practitioner. S = session. SOC = Stage of Change questionnaire. T = time. TNM = tumour, node, metastases.*

### Reach

The number of invited and participating patients, and reasons for not inviting patients to participate were documented by PCPs. To evaluate representativeness, characteristics of participants and non-participants were collected using questionnaires (Box 1). Reasons for programme (non-)participation were collected via self-report.

### Effectiveness

#### Primary outcomes

Fatigue was measured using the Functional Assessment of Cancer Therapy — Fatigue (FACT-F) questionnaire.^42^ A three-point difference signifies a minimal clinically important difference (MCID).^43^ Depression and anxiety symptoms were assessed using the Hospital Anxiety and Depression Scale (HADS),^44^ with a 1.5-point difference as MCID.^45^^,^^46^

#### Secondary outcomes

Daily step count and caloric expenditure of the week before each coaching session were extracted from the activity tracker by the PCP. A 784-step increase was considered an MCID.^47^ Lower-limb strength and aerobic endurance were assessed by the PCP with the 30-s sit-to-stand test and the 2-min step test, with a two-count and a nine-count improvement indicating an MCID, respectively.^48^^–^^51^ Self-reported PA was measured by the International Physical Activity Questionnaire — Short Form (IPAQ-SF).^52^ Quality of life was assessed with the Function Assessment of Cancer Therapy — General (FACT-G),^53^ with a four-point difference signifying an MCID.^43^ Programme experiences were assessed through self-report questionnaires. Participants rated if the programme increased their energy, if the activity tracker helped achieve their PA goals, and if they would recommend the programme.

### Implementation

Individuals who dropped out and their reasons were documented by the PCP. Session adherence and PA goal accomplishment were recorded by the PCP. Adherence to activity tracker use was evaluated via self-report.

### Data analyses

The RE-AIM dimensions were analysed descriptively, presented as means and standard deviations (SDs), numbers and percentages, or medians and interquartile ranges. Participation rate was calculated based on the number of invited and participating patients. Characteristics of participants and non-participants were compared using independent *t*-tests or non-parametric tests. Patient characteristics were compared between those who dropped out and those who continued participation. PA goal achievement during sessions S2–S4 was averaged and deemed successful if patients met ≥90% of their goal. Programme experiences were averaged from T_1_ to T_3_. Adherence to counselling sessions was calculated as the number of attended sessions relative to the total sessions a patient participated in the programme (for example, for patients who completed the entire programme adherence was assessed up to the final session, while for those who discontinued earlier it was measured up to their dropout point).

Linear mixed-model analyses were employed to evaluate changes in primary and secondary outcomes over time, incorporating three levels: general practice, patient, and measurement time. This approach enabled the inclusion of missing data in the analysis by accounting for variations in the number of repeated measurements per individual, assuming the data were missing at random.^54^ Random intercepts were included at the practice and patient levels to account for variations between settings and individuals. Time was treated as a fixed effect categorical variable, with baseline (T_0_/S1) as the reference. Normality and homogeneity of error variances were verified using histograms, Q–Q plots, and scatterplots. If residuals were not normally distributed, the authors log-transformed the outcome variable and used the transformed data when assumptions were met. Parameter estimates were then back-transformed to the original scale. Non-converging models had the random intercept for general practice removed. Outcome changes were illustrated using graphs with confidence interval bars, and changes compared with the MCID, if available. Data analysis was performed using IBM SPSS Statistics (version 23).

## Results

### Reach

#### Patient inclusion

**Figure 1.**
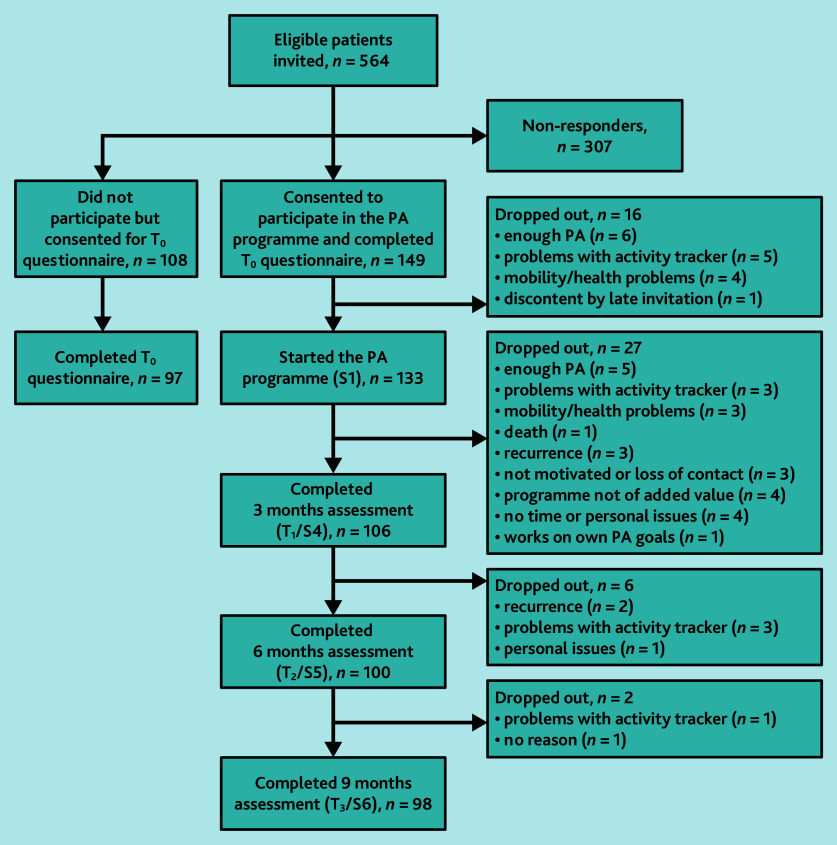
Flowchart of patient recruitment. PA = physical activity. S = session. T = time.

In total, 3250 patients with a cancer diagnosis were identified at 15 general practices (see Supplementary Figure S1). In 10 practices, PCPs screened 1528 patients for eligibility; 484 patients were excluded based on study criteria and 412 based on PCPs’ judgement. Their reasons for excluding patients were most commonly old age (*n* = 230, 56%) and a cancer diagnosis too long ago (*n* = 44, 11%). In total, 564 eligible patients were invited; 149 enrolled in the programme (26%, range practices 0%–71%), 108 chose not to participate (that is, non-participants), and 307 did not respond (Figure 1).

#### Patient characteristics

Participants had less formal education (*P* = 0.010), were more often unemployed (*P* = 0.012), less often alcohol consumers (*P* = 0.041) with less alcohol consumption (*P* = 0.014), and had lower total and moderate activity (*P* = 0.012 and 0.042, respectively) metabolic equivalent of tasks (METs) min/week compared with non-participants at baseline (Table 1). Additionally, participants reported more fatigue (*P*<0.001), anxiety (*P* = 0.002), and depression (*P* = 0.011) symptoms, and lower quality of life scores (*P*<0.001).

**Table 1.** Baseline characteristics of participants and non-participants

**Characteristic**	**Participants, *n* = 149^ab^**	**Non-participants, *n* = 108^ab^**	***P*-value**
**Demographic information**			
Age, years, mean (SD)	63.7 (11.4)	65.7 (9.3)	0.058
Sex, female	100 (69)	59 (59)	0.126
Formal education			0.010
Short	27 (19)	17 (17)	
Medium	71 (49)	32 (32)	
Long	47 (32)	51 (51)	
Employment status			0.012
Employed	40 (29)	34 (34)	
Unemployed	43 (31)	14 (14)	
Retired	57 (41)	51 (52)	
Living situation			0.897
With others	112 (77)	76 (76)	
Single	34 (23)	24 (24)	
Care received at home			0.863
Yes	8 (5)	6 (6)	
No	138 (95)	94 (94)	

**Lifestyle measures**			
Smoking			0.075
Never	58 (43)	30 (30)	
Ever	59 (44)	58 (59)	
Current	18 (13)	11 (11)	
Pack years,^c^ median (IQR)	11 (4–25)	15 (5–30)	0.226
Alcohol			
Current consumer	74 (55)	68 (68)	0.041
Units per week,^c^ median (IQR)	4 (1–7)	5 (3–10)	0.014
SOC			0.208
1. Precontemplation	2 (1)	5 (5)	
2. Contemplation	8 (6)	6 (6)	
3. Preparation	26 (19)	15 (15)	
4. Action	30 (22)	14 (14)	
5. Maintenance	69 (51)	60 (60)	
IPAQ-SF, METs min/week, median (IQR)			
Walking	693 (380–1485)	891 (396–1782)	0.438
Moderate activities	600 (0–1440)	960 (240–1965)	0.042
Vigorous activities	0 (0–480)	0 (0–1680)	0.061
Total	1760 (581–4212)	2589 (1230–4883)	0.012
Sitting (min/weekday)	360 (240–480)	300 (240–480)	0.191

**Clinical characteristics**			
Cancer diagnosis			0.542
Breast	33 (23)	22 (23)	
Colorectal	17 (12)	8 (8)	
Melanoma	14 (10)	4 (4)	
Haematological	11 (8)	5 (5)	
Bladder	10 (7)	6 (6)	
Prostate	8 (6)	6 (6)	
Multiple	26 (18)	21 (22)	
Other	23 (16)	23 (24)	
Cancer stage^d^			0.944
Local	71 (64)	48 (62)	
Locally advanced	36 (32)	27 (35)	
Metastatic	4 (4)	3 (4)	
Recurrence	16 (11)	12 (11)	0.735
Type of treatment			
Surgery	121 (81)	77 (71)	0.062
Chemotherapy	60 (40)	36 (33)	0.257
Radiotherapy	68 (46)	43 (40)	0.352
Immune therapy	13 (9)	12 (11)	0.524
Hormone therapy	31 (21)	21 (19)	0.789
Adjuvant hormone therapy	13 (9)	9 (8)	0.707
Other	5 (3)	6 (6)	
Time since diagnosis, years, median (IQR)	5 (2–10)	6 (3–12)	0.239
Time since treatment, years, median (IQR)	4 (2–9)	6 (3–11)	0.108
Comorbidity index, median (IQR)	2 (2–3)	2 (2–3)	0.217

**Health outcomes, mean (SD)**			
Fatigue (FACT-F)	37.7 (10.3)	44.1 (8.5)	<0.001
Anxiety (HADS-A)	4.6 (3.6)	3.2 (2.9)	0.002
Depression (HADS-D)	3.9 (3.4)	2.8 (3.2)	0.011
QoL (FACT-G)	81.3 (13.0)	87.1 (12.8)	<0.001

a *Data are* n *(%) unless otherwise indicated.*

b *There are missing data for some characteristics.*

c *Calculated for consumers only (that is, ever and current smokers/alcohol consumers).*

d *Reported for solid tumours only; local: T1-2N0M0, locally advanced: T1-2N1M0/T3-4N0-1M0, and metastatic: T1-4N0–1M1.* N*-values for participants and non-participants equal 111 and 78, respectively. FACT-F = Functional Assessment of Cancer Therapy — Fatigue. FACT-G = Functional Assessment of Cancer Therapy — General. HADS-A = Hospital Anxiety and Depression Scale — Anxiety. HADS-D = Hospital Anxiety and Depression Scale — Depression. IPAQ-SF = International Physical Activity Questionnaire — Short Form. IQR = interquartile range. METs = metabolic equivalent of tasks. QoL = quality of life.*

*SD = standard deviation. SOC = Stage of Change questionnaire. TNM = tumour, node, metastases.*

Main reasons for joining the PA programme included health benefits (*n* = 111; 74%), improving physical fitness (*n* = 101; 68%), reducing fatigue (*n* = 71; 48%), or losing weight (*n* = 59; 40%). Non-participants primarily cited being already physically active (*n* = 74; 69%) as a reason for not joining (see Supplementary Figure S2).

### Effectiveness

#### Primary outcomes

**Figure 2.**
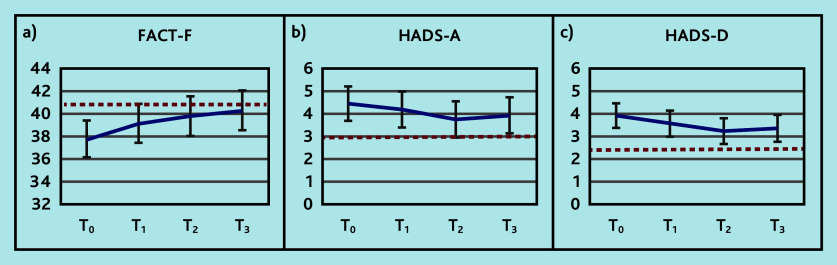
Trajectory in the primary outcomes: a) FACT-F (range 0–52, higher score better outcome); b) HADS-A (range 0–21, lower score better outcome); and c) HADS-D (range 0–21, lower score better outcome) at time (T)_0_ (baseline), T_1_ (3 months), T_2_ (6 months), and T_3_ (9 months). The horizontal dashed red line indicates the minimal clinically important difference relative to baseline. FACT-F = Functional Assessment of Cancer Therapy — Fatigue. HADS-A = Hospital Anxiety and Depression Scale — Anxiety. HADS-D = Hospital Anxiety and Depression Scale — Depression.

Reductions from baseline were observed for all the primary outcomes, FACT-F, HADS-A, and HADS-D, especially until the third measurement point (Figure 2 and Supplementary Table S1). These reductions were below the MCID.

#### Secondary outcomes

**Figure 3.**
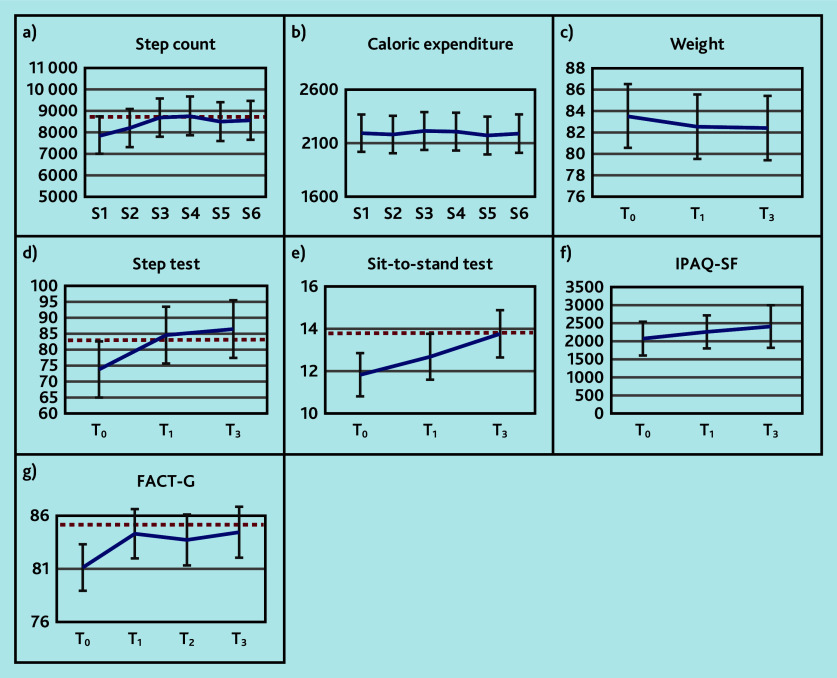
Trajectories of secondary outcomes: a) step count; b) caloric expenditure (kcal); c) weight (kg); d) step test; e) sit-to-stand test; f) IPAQ-SF (total METs min/week); and g) FACT-G (range 0–108) at time (T)_0_ (baseline), T_1_ (3 months), T_2_ (6 months), and T_3_ (9 months) or at session (S)1 (baseline), S2 (3 weeks), S3 (6 weeks), S4 (3 months), S5 (6 months), and S6 (9 months). Higher scores represent better outcomes. The horizontal dashed red line indicates the minimal clinically important difference relative to baseline. FACT-G = Functional Assessment of Cancer Therapy — General. IPAQ-SF = International Physical Activity Questionnaire — Short Form. MET = metabolic equivalent of task.

All secondary outcome measures of activity and fitness (step count, IPAQ-SF, step test, and sit-to-stand test) increased over time, except for caloric expenditure, which remained constant (Figure 3 and Supplementary Table S1). Weight decreased on average over time. These differences exceeded the MCID for some of the time points for step count, step test, and 30-s sit-to-stand test; for the other secondary outcomes they were below the MCID.

#### Programme experiences

On average across the time points T1 to T3, 35% (*n* = 34/98) of participants reported a high increase in energy, 53% (*n* = 52/98) noticed a moderate or slight increase, and 12% (*n* = 12/98) reported no change. Regarding PA, 39% (*n* = 39/99) reported a high increase, 46% (*n* = 46/99) moderate or slight, and 14% (*n* = 14/99) no increase (see Supplementary Figure S3).

Nearly half of the participants (46%; *n* = 45/97) found the activity tracker highly helpful in achieving their PA goals, 44% (*n* = 43/97) found it somewhat helpful, and 9% (*n* = 9/97) found it unhelpful. Additionally, two-thirds (66%; *n* = 65/99) would highly recommend the programme to others, nearly one-third (31%; *n* = 31/99) would recommend it to some extent, and 3% (*n* = 3/99) not at all (see Supplementary Figure S3).

### Implementation

Overall, 16 of the 149 consenting patients (11%, range practices 0%–40%) dropped out before the programme began, and 35 of the remaining 133 (26%, range practices 0%–67%) dropped out during the programme. Most cited reasons for dropping out were issues with the activity tracker (*n* = 12) and feeling sufficiently physically active (*n* = 11) (Figure 1). Those who dropped out were more often employed, had less advanced cancer, and reported lower total METs min/week than those who continued (see Supplementary Table S2).

Adherence to the counselling sessions on the time point patients participated in the programme was high (98%; *n* = 647/661); 92% (*n* = 92/100) of patients wore the activity tracker 1 week before each coaching session. In the other weeks of the programme, 83% (*n* = 83/100) wore it daily. Twelve patients used their own tracker. On average, 73% met their PA goals during S2–S4 (S2: 67%, *n* = 81/121; S3: 81%, *n* = 91/112; and S4: 71%, *n* = 70/99) (data not shown).

## Discussion

### Summary

One in four eligible cancer survivors participated. Participants had less formal education, higher unemployment rates, were less physically active, consumed less alcohol, and experienced more fatigue and psychological symptoms than non-participants. Over time, favourable but sub-MCID changes were observed for fatigue, anxiety, and depressive symptoms. Favourable changes were observed for most secondary outcomes, with step count and physical function reaching MCID. Many participants reported increased PA and energy. Despite a 26% dropout rate of those who started the PA programme, primarily because of difficulties with the activity tracker or experiencing sufficient PA, programme adherence was high.

### Strengths and limitations

This study’s strengths include detailed documentation of the inclusion process, broad selection criteria, and the comparison with non-participants. This enabled comprehensive data collection about the proportion and characteristics of cancer survivors reached by the programme, reflecting a real-world setting. Also, it was possible to include long-term cancer survivors with various diagnoses whereas most studies included short-term survivors of breast cancer.^12^^,^^14^^,^^55^ A limitation is the absence of a control group, preventing definitive attribution of health changes solely to the PA programme. However, this study aimed to gather insights on the implementation of PA programmes in primary care, rather than to provide evidence of effectiveness, which is already well established.^10^^–^^15^ Additionally, the authors regard it unlikely that the observed health changes are owing to time, given that participants were long-term survivors with minimal expected recovery in the short study period.

### Comparison with existing literature

The current programme contrasts with previous studies typically involving participants with more formal education, who are more active, and with less psychological symptoms,^23^^–^^25^ suggesting this study reached a group that stands to benefit the most. Notably, the participants were long-term survivors (averaging 5 years post-diagnosis), with half seeking relief from fatigue — unlike most PA studies that have focused on survivors within 1–5 years post-diagnosis.^14^^,^^55^ This highlights unmet care needs among long-term survivors, as up to 50% experience enduring fatigue.^2^^,^^56^^,^^57^ Unlike most PA studies, the current study did not restrict eligibility based on PA levels or symptom severity. As such, it may have engaged a relatively healthy cohort, as many non-participants and those who dropped out felt they were already sufficiently active. This may explain the lower participation and higher dropout rates compared with previous studies.^14^^,^^21^^,^^58^ Additionally, this likely resulted in relatively low FACT-F and HADS baseline scores compared with previous PA studies,^59^^–^^65^ leaving little room for improvement. Nevertheless, the current findings suggest that this programme may alleviate these symptoms even in survivors with low symptom severity. This is exemplified by the finding that participants reported a perceived increase in energy, supported by the study’s qualitative findings.^36^ The programme appears particularly effective in improving PA and physical function, reflected by the clinically relevant changes, consistent with previous research.^66^^–^^68^ The sustained step count increase after the third and fourth sessions shows the programme’s effectiveness in maintaining higher activity levels. The use of activity trackers and counselling sessions in the current programme may have contributed to increased PA as participants found these elements highly motivating,^36^ aligning with previous literature.^69^ Activity trackers alone may increase PA among cancer survivors;^70^^–^^72^ combining them with feedback and coaching enhances adherence, PA behaviour, and health outcomes.^14^^,^^67^^,^^72^^,^^73^ Coaching strategies like self-monitoring, problem solving, and fostering autonomy and motivation add to PA maintenance,^74^^,^^75^ which is often limited when relying solely on activity trackers.^9^^,^^70^ However, digital challenges with tracker use, as observed in the reasons for dropping out among participants in the current study, highlight the need for technical support.

### Implications for research and practice

These findings highlight an important role that PCPs can play in the symptom control and care needs of cancer survivors. A PA programme in primary care can enhance cancer survivors’ activity levels and health outcomes, regardless of time post-diagnosis. However, not all survivors may need this intervention; many manage their symptoms independently or do not need care. The authors recommend PCPs target these programmes at those with significant needs, such as fatigue and low PA levels, especially given the high pressure in primary care. GPs could incorporate their practice nurses in providing this care, but may also advise patients to search for other community activities or refer them to other healthcare professionals or PA programmes based in the community. The combination of activity trackers and coaching sessions appears especially effective, and could be easily adopted for other chronic conditions like diabetes and pulmonary diseases.

Future research may use stricter eligibility criteria (low PA and high symptom severity) and include longer follow-up periods to assess long-term outcomes.

To conclude, the current study showed that a home-based PA programme, with PCP counselling and activity tracker use, reached long-term cancer survivors with poorer health status and shows favourable health changes particularly on PA and physical function. Such PA programmes may benefit the health of a rising number of cancer survivors visiting primary care.

## Data

The datasets analysed during the current study are available from the corresponding author on reasonable request.
